# An integrated model to evaluate the impact of social support on improving self-management of type 2 diabetes mellitus

**DOI:** 10.1186/s12911-019-0914-9

**Published:** 2019-10-22

**Authors:** Xiaojia Wang, Linglan He, Keyu Zhu, Shanshan Zhang, Ling Xin, Weiqun Xu, Yuxiang Guan

**Affiliations:** 1 Department of Information Management, School of Management, Hefei University of Technology, Mailbox 270, No. 193, Tunxi Road, Hefei, 230009 An Hui Province China; 2 Department of Clinical Teaching, The First Affiliated Hospital of Anhui University of Chinese Medicine, Hefei, 230031 Anhui China; 3 The National Chinese Medicine Clinical Research Base- Key Disease of Diabetes Mellitus Study, Hefei, 230031 Anhui China; 4 Healthcare and Public health Information Center, The First Affiliated Hospital of Anhui University of Chinese Medicine, Hefei, 230031 Anhui China; 5 Department of Endocrinology, The First Affiliated Hospital of Anhui University of Chinese Medicine, Hefei, 230031 Anhui China

**Keywords:** Type 2 diabetes, Self-management, Social support, Evaluation indicator system, ANP, CRITIC

## Abstract

**Background:**

Type 2 Diabetes Mellitus (T2DM) is a chronic disease closely related to personal life style. Therefore, achieving effective self-management is one of the most important ways to control it. There is evidence that social support can help to improve the self-management ability of patients with T2DM, but which social support is more effective has been rarely explored. The purpose of this study is to construct an integrated model to analyze which social support has more significant impact on self-management of T2DM, and provide reasonable suggestions to health care providers on how to effectively play the role of social support.

**Methods:**

We established a social support indicator evaluation system and proposed an integrated model that combines ANP (Analytical Network Process) and CRITIC (CRiteria Importance through Intercriteria Correlation) methods to evaluate the impact of social support on T2DM self-management from both subjective and objective perspectives. The weights calculated by the model will serve as the basis for us to judge the importance of different social support indicators.

**Results:**

Informational support (weighting 49.26%) is the most important criteria, followed by tangible support (weighting 39.24%) and emotional support (weighting 11.51%). Among 11 sub-criteria, guidance (weighting 23.05%) and feedback (weighting 14.68%) are two most relevant with T2DM self-management. This result provides ideas and evidence for health care providers on how to offer more effective social support.

**Conclusion:**

To our knowledge, this is the first study in which Multi-Criteria Decision Making (MCDM) tools, specifically ANP and CRITIC, are used to evaluate the impact of social support on improving self-management of type 2 diabetes. The study suggests that incorporating two sub-indicators of guidance and feedback into the diabetes care programs may have great potential to improve T2DM self-management and further control patient blood glucose and reduce complications.

## Background

The risk and prevalence of diabetes mellitus has dramatically increased over the past decade [1, 2]. According to the International Diabetes Federation (IDF), the number of individuals affected by diabetes was over 425 million in 2017 and is predicted to surge to 642 million by 2040 [3]. Moreover, most of these cases are type 2 diabetes. T2DM has become a serious threat to people’s health.

Research has shown that unhealthy lifestyles including overweight or obesity, insufficient physical activity, smoking, and unhealthy dietary practices can greatly increase the occurrence of type 2 diabetes and related complications [4] . Effective self-management can help improve T2DM health outcomes by modifying these unhealthy lifestyles. There is some evidence to suggest that the self-management of T2DM is the cornerstone to achieving good glycemic control and reducing the risk of developing microvascular (retinopathy, nephropathy, and neuropathy) and macrovascular (cardiovascular and cerebrovascular disease) complications [5]. Therefore, it is crucial to improve the self-management capability of patients with type 2 diabetes [6].

However, for various reasons, it is very difficult to rely on the patients themselves to realize T2DM self-management. Clinicians often rely on medical management and self-management of patients themselves to manage diabetes control. Research indicates that good diabetic health may not be sustainable because psychosocial factors hinder the best practice of self-management of diabetes [5]. Investigating the factors most relevant to T2DM self-management, we find that social support can contribute to the successful management of the disease [7]. It has been shown to be positively related to health behavioral change in chronic illness management, particularly in the field of diabetes [8–11]. People who frequently interacted with other diabetics showed lower levels of HbA1c (Glycosylated Hemoglobin, a measure of glycemic control) than those who lacked social support [12]. Furthermore, people have better diabetes outcomes when involved in diabetes self-management education programs that combine behavioral and psychosocial strategies (e.g., social support) [13]. Therefore, strategies that promote diabetes self-management should include social support [14].

The positive impact of good social support on self-management is widely recognized in public health research [15]. However, there are still few studies on which dimensions of social support are more important for influencing self-management. Diabetes research in China, a country with the highest diabetes prevalence in Asia and the largest absolute disease burden of diabetes worldwide [16, 17], has focused mainly on diagnosis and treatment, neglecting the role of social support [18]. Therefore, this article aims to analyze which dimensions of social support have greater impact on T2DM self-management and explore coping strategies to enhance patients’ self-management. In order to visualize the importance of different social support indicators, we proposed the ANP (Analytic Network Process)–CRITIC (CRiteria Importance through Intercriteria Correlation) model to quantify the indicators weights. On the one hand, we invited experts to rate the importance of indicators, and converted the expert scores into subjective weights through the ANP method; on the other hand, we recruited 3000 patients with type 2 diabetes to fill out the Social Support Measurement Questionnaire we developed and converted the results into objective weights through the CRITIC method. And we finally obtained the comprehensive weights by the fusion of the least squares method. The greater the weight of the indicator, the more important it is to improve the self-management of type 2 diabetes. The results will be decisive for both the development and management of T2DM and help health care providers focus their attention on the most rewarding social support dimensions.

## Methods

### The evaluation indicator model

Social support was an important explanatory variable with prognostic significance for health outcomes [5]. It was defined as help provided by family, friends, neighbors, or others and included different domains, such as information, emotional comfort, and practical help [19]. Although there were many different social support dimensions, we surveyed more than 1000 articles related to social support and diabetes in which the appearance of “emotional support,” “information support” and “tangible support” accounted for 90, 88, and 83%, respectively, much higher than other social support dimensions. In fact, whether from the different definition of social support or from the related research of social support, emotional, informational, and tangible support were the three most widely recognized dimensions of social support [19–22]. In view of the analysis, we used the three dimensions to build a social support indicator system to comprehensively analyze the influence of social support on the self-management of T2DM.

As for the selection of sub-criteria, we first pre-screened them based on the definition of their control criteria and T2DM-related literature, and then decided by the expert panel through discussion. The panel included 15 experts with extensive experience in T2DM self-management, who were from the KDM ((Key Disease of Diabetes Mellitus Study) (six people), the Chinese Academy of Diabetes Science (five people), and the First Affiliated Hospital of the University of Science and Technology of China (four people). Under each control criteria (emotional support, informational support, and tangible support), we finally identified several sub-criteria to support the evaluation.

#### Emotional support (ES)

The distress associated with diabetes was a common emotional state among patients with T2DM. Type 2 diabetes was a complex and lifelong chronic disease that required a lot of self-management on the part of the individual. As a result, many people with type 2 diabetes might feel overwhelmed and depressed by a range of multifaceted and often required self-care activities, as well as by the threat of long-term complications. Emotional support included providing empathy, care, love and trust. This kind of support could enhance the sense of self-worth and affirmation, and the coping efforts of patients with T2DM [23]. We included four sub-criteria to measure emotional support: (i) Encouragement support (E1), (ii) Listening support (E2), (iii) Express respect (E3), and (iv) Empathetic understanding (E4).

#### Informational support (IS)

Informational support referred to providing guidance, advice, and counseling to those under stress. Such support might help individuals benefit from advice on how to best respond to the challenges of type 2 diabetes [23]. Patients with T2DM usually needed information on their condition and actions they should take. They also required some feedback about what they were doing. Therefore, we included three sub-criteria to measure informational support: (i) Status analysis of the condition (I1), (ii) Guidance (I2), and (iii) Feedback (I3).

#### Tangible support (TS)

Tangible support was the “provision of tangible goods and services or tangible aid.” It referred to offering material aid or behavioral assistance [24]. Tangible support could be measured by (i) Healthy food (T1), (ii) Physical activity (T2), (iii) Medicine and medical instruments (T3), and (iv) Financial support (T4). This kind of support might be necessary for T2DM patients to perform diabetes self-management behaviors [23].

**Fig. 1:**
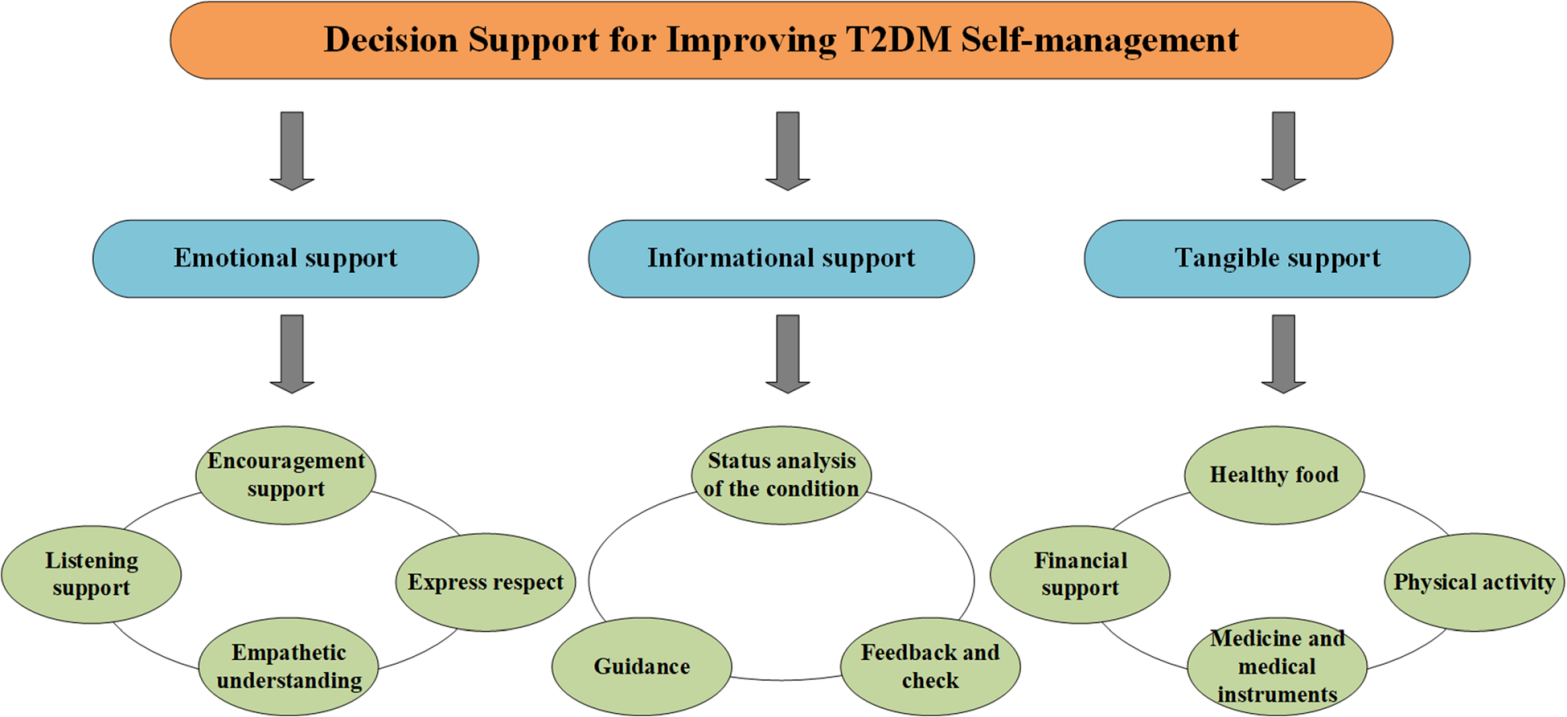
Hierarchy of criteria. Hierarchy of criteria: Based on literature review and inputs from experts, we established a reasonable indicator system of social support consisting of three criteria and 11 sub-criteria. The control criteria include emotional support, informational support and tangible support, which are the three most widely recognized social support dimensions to benefit the self-management of type 2 diabetes. As for the selection of sub-criteria, we first pre-screened them based on the definition of their control criteria and T2DM-related literature, and finally decided by the expert panel

Under each of the three control criteria, 11 sub-criteria were developed (Fig.1); the details were in Additional file 1.

Based on the above selected criteria, we described the weighting methods to determine their subjective and objective weights in the following sections 2.2 and 2.3 respectively. In section 2.4, we achieved the fusion of subjective weight and objective weight by least square method, and obtained the comprehensive weights of indicators. In order to present the importance of indicators more intuitively, we further divided the indicators into three levels using the rank-sum ratio (RSR) method in section 2.5.

### Subjective weighting method: ANP

The Analytic Network Process (ANP) [25], an extension of the Analytic Hierarchy Process (AHP) [26, 27], was a comprehensive decision-making technique that could include all the relevant criteria to arrive at a decision. In the ANP, a complicated decision problem was decomposed into a rational decision hierarchy based on related attributes or criteria. The ANP could resolve complex multi-criteria decision problems when they involved multi-criteria or hierarchy dependence relationships.

The ANP’s stepwise algorithm used in this study was stated by Saaty [25] as follows:

- Step 1: Describe the decision problem in detail with the goal, criteria, and sub-criteria.
- Step 2: Determine the general network of components/clusters and the elements within the clusters.
- Step 3: Determine all inter- and inner-dependencies that exist in the decision problem and the clusters of the general feedback system according to the expert panel.
- Step 4: Build the super-matrix by performing the pairwise comparisons and prioritization, and define the weights of the control criteria and the sub-criteria while considering the interdependencies between them.

To make it easier to understand, the key steps were described in more detail below.

**Fig. 2:**
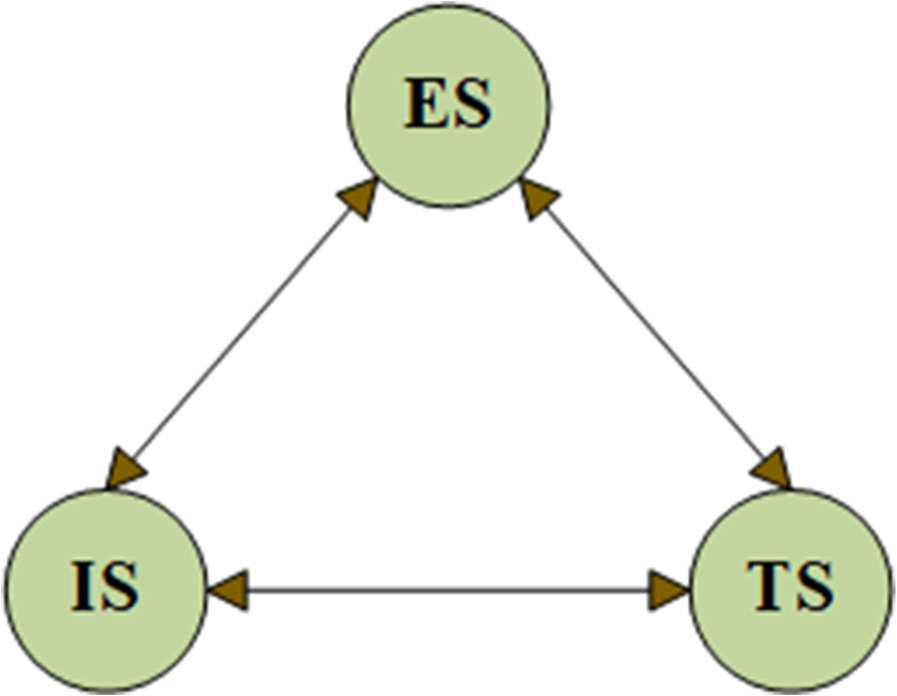
Control criterion internal influence relationship. Control criterion internal influence relationship: It showed the correlation between the three control criteria, which included the ES (Emotional support), IS (Informational support) and TS (Tangible support). Experts believed that there was a mutual relationship between each two of them

**Fig. 3:**
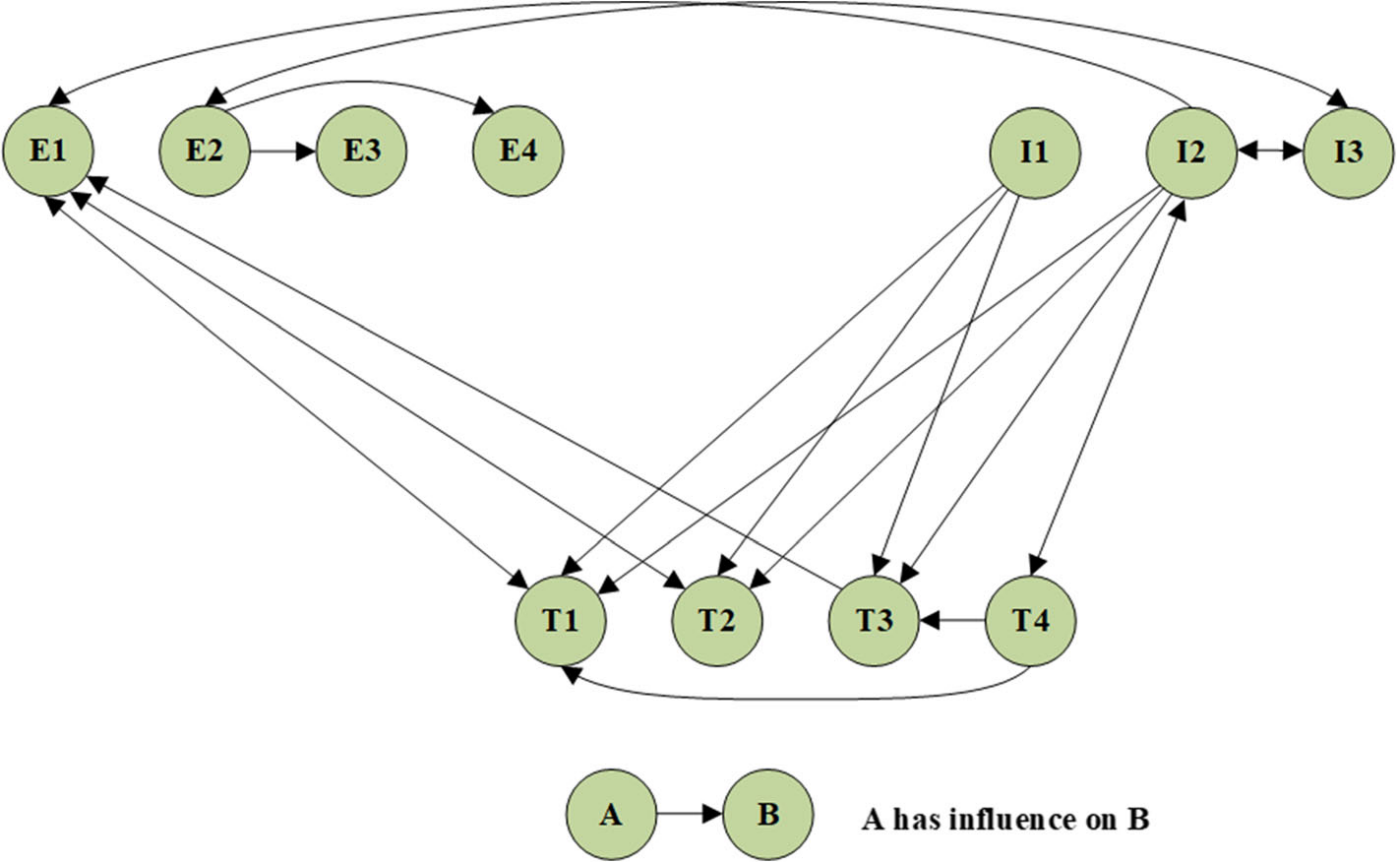
The relationship between the sub-criteria. The relationship between the sub-criteria: It showed the correlation between the 11 sub-criteria, which included E1 (Encouragement support), E2 (Listening support), E3 (Express respect), E4 (Empathetic understanding), I1 (Status analysis of the condition), I2 (Guidance), I3 (Feedback), T1 (Healthy food), T2 (Physical activity), T3 (Medicine and medical instruments), T4 (Financial support). Experts believed that some sub-criteria had a certain relationship and had been marked with arrows

Firstly, we determined all interdependencies between the control criteria and sub-criteria according to the expert panel. Considering the correlation between the criteria proposed by the panel, we built the relationship between the control criteria (Fig. 2) and correlations between the sub-criteria (Fig. 3).

Secondly, we constructed pairwise comparison matrices through expert judgement. It was difficult to assign weights to each indicator directly, but it was relatively easy to compare the importance between two indicators. Therefore, experts were asked to score the relative importance of two factors according to Saaty’s 1–9 scale (for details, see Additional file 2). After repeated face-to-face discussions, the experts reached a consensus. Now the value *a*_*ij*_ obtained by comparing two factors into the position of the *jth* column of the *ith* row of the matrix. A reciprocal value was assigned to the inverse comparison, that was, *a*_*ij*=_1/*a*_*ji*_, where *a*_*ij*_(*a*_*ji*_) denoted the importance of the *ith*(*jth*) element, and a comparison matrix was constructed. The diagonal line was 1, because it was the ratio of oneself to oneself. Like AHP, pairwise comparison in ANP was made in the framework of a matrix, and a local priority vector could be derived as an estimate of relative importance associated with the elements (or components) being compared by solving the following equation:

1 $$ AW={\lambda}_{\mathrm{max}}W $$

where *A* was the comparison matrix. *W* was the eigenvector, and *λ*_max_ was the largest eigenvalue of *A* . Hence, *A* was consistent if and only if *λ*_max_ = *n*. It was suggested to normalize obtained vectors to sum of each one becomes one. The eigenvector *W* corresponding to the largest eigenvalue *λ*_max_ of matrix *A* was the weight vector we wanted to get after normalization.

In the process of constructing a comparison matrix, it should be noted that the expert’s judgment preference must be tested by consistency. The measure of consistency of judgment was measured through the Consistency Index (CI) and Consistency Ratio (CR). The CI, as a measure of degree of consistency, was calculated using the formula:

2 $$ CI=\frac{\lambda_{\mathrm{max}}-n}{n-1} $$

The consistency ratio (CR) was computed as

3 $$ CR=\frac{CI}{RI} $$

where RI was the mean random consistency index. Acceptable CR values must be less than 0.1. Decision-makers were asked to repeat the pairwise comparisons for CR values greater than 0.1 [28].

Thirdly, we built the super-matrix after considering the correlations between the sub-criteria. Then we multiplied super-matrix by the weight vector of the sub-criteria to obtain the final subjective weights of indicators.

### Objective weighting method: CRITIC

The CRiteria Importance through Intercriteria Correlation (CRITIC) proposed by Diakoulaki was a method of determining objective weights that was based on the quantification of two fundamental notions of Multi-Criteria Decision-Making (MCDM): the contrast intensity and the conflicting character of the evaluation criteria [29]. The extraction and exploitation of these two features that were stored as intrinsic information in the data defining the multi-criteria problem were beneficial to the decision-making process. Objective weights derived by this method incorporated both the contrast intensity of each criterion and the conflict between the criteria. The contrast intensity of the criteria was considered by the standard deviation and the conflict between them was measured by the correlation coefficient [30].

CRITIC’s stepwise algorithm used in this study was as follows:

Step 1: Calculate the transformed values of sub-criteria and obtained the criteria vectors as follows:

4 $$ {x}_{aj}=\frac{f_j(a)-{f}_{j^{\ast }}}{f_j^{\ast }-{f}_{j^{\ast }}} $$

where *x*_*aj*_ expressed the degree to which the alternative *a* was close to the ideal value ${f}_j^{\ast }$, which was the best performance in criterion *j*, and far from the anti-ideal value ${f}_{j^{\ast }}$, which was the worst performance in criterion *j*. Both ${f}_j^{\ast }$ and ${f}_{j^{\ast }}$ were achieved by at least one of the alternatives under consideration.

Step 2: Calculate the standard deviation (*σ*_*j*_) of each vector (*x*_*j*_).

Step 3: Construct correlation coefficient matrices, with dimension *m* × *m* and generic elements *r*_*jk*_.The elements of this matrix were the linear correlation coefficient between the vectors *x*_*j*_ and ${x}_{k^{\hbox{'}}}$. It should be noted that if all elements of *x*_*j*_ and *x*_*k*_ vectors were identical, we could suppose that there was no correlation (*r*_*jk*_ = 0).

Step 4: Calculate the information measures of each criterion as follows:

5 $$ {C}_j={\sigma}_j\sum \limits_{k=1}^m\left(1-{r}_{jk}\right) $$

Step 5: Objective weights resulted by normalizing these values to unity according to the eq. (6):

6 $$ {w}_j=\frac{C_j}{\sum \limits_{k=1}^m{C}_k} $$

To get the raw experimental data required in Step 1, we recruited 3000 patients diagnosed with T2DM from the KDM to assist filling out the Social Support Measurement Questionnaire developed by our team.

The recruited patients were in the Anhui Provincial Hospital of Traditional Chinese Medicine from February 2016 to February 2018, which was one of the two selected diabetes research bases that conducted the KDM and aimed to explore new methods of diabetes prevention and care through a quantized analysis. The diagnostic criteria of type 2 diabetes developed by the WHO (World Health Organization) in 2006 [31] were used, and the participants were all aware and had a certain understanding regarding the study. Before the start of the experiment, each person had carefully read the informed consent form of the experiment and signed to volunteer to participate in the experiment (for details, see Additional file 3). Then they were asked to fill out the Social Support Measurement Questionnaire (for details, see Additional file 4) as required. Information about their received social support and HbA1c was collected by the diabetes care clinic in the KDM.

The Social Support Measurement Questionnaire’s design took into account the needs of our research itself and three widely used social support questionnaires: (1) The Inventory of Socially Supportive Behaviors (ISSB), one of the most commonly used measure, consisted of 40 questions that specifically asked respondents about the frequency of emotional support, informational support, and practical support received from any (unspecified) network members recently [32]. A study of 200 Chinese college students in Beijing and Nanjing showed that the translated ISSB had a Cronbach’s alpha of 0.94 (The Cronbach alpha is one of the most commonly used statistics for measuring the reliability of a questionnaire. When it reaches 0.8–0.9, the reliability of the scale is very good) [33]. (2) The Social Provisions Scale (SPS) based on Weiss’s [34] model of social provisions contained 24 items inquiring about the frequency of recent receipt of practical help, informational support, emotional support, esteem support, social integration, and opportunity to provide nurturance [35]. (3) The Medical Outcomes Study (MOS) Social Support Survey was another valid and reliable measure of social support that had demonstrated test-retest reliability and internal consistency reliabilities greater than 0.91 [36]. We selected the appropriate questions from the three scales and adjusted them to ensure that each indicator in the system we established would be measured by 3 questions. Then the Social Support Measurement Questionnaire implemented by our research was obtained. Finally, there were 2969 participants remaining after the removal of 31 participants whose questionnaires were incomplete.

For each question in the Social Support Survey Questionnaire, when the respondents chose “Strongly Agree “, “ Agree “, “ Not Sure “, “ Disagree “ or “ Strongly Disagree “, they will get 9, 7, 5, 3 and 1 points respectively. This meant that after each patient completed the questionnaire, the score result would be an 11-dimensional vector, which correspondingly showed the score of 11 sub-criteria the patient got (each sub-criteria was measured by three questions, and the score of each sub-criterion was added up to get the score of the sub-criteria). Considering patients’ lack of self-awareness or misunderstanding of the problem, we also invited the patient’s attending physician to revise each score according to the actual situation of the patient they knew and got the final score. (Full score 10, minimum score 0. The higher the score, the better social support the patient received.). In this way, we translated the social support scores of 2969 patients into 2969 11-dimensional vectors.

### Integrating ANP and CRITIC: least squares

As classic multi-criteria decision-making methods, we can get credible subjective and objective weights from ANP and CRITIC methods respectively. The subjective weighting method ANP emphasized the evaluation judgment for the criterion, while the objective weighting method CRITIC focused on the underlying information of each criterion. In order to achieve the unity of objectivity and subjectivity, we adopted a linear combination weighting approach - Least Squares- to integrate ANP and CRITIC and got the comprehensive weights.

### Indicators classification method: RSR

RSR was a statistical analysis method that integrated the advantages of classical parameter estimation and modern non-parametric estimation [37]. It was a powerful and promising statistical analysis research tool for mathematical modeling of operational processes and had been successfully applied to various decision-making activities in various fields [38].

We used this method to classify the indicators into levels with different importance on improving T2DM self-management, and gave corresponding suggestions to benefit patients, which would be elaborated in section 3.3 and 3.4.

**Fig. 4:**
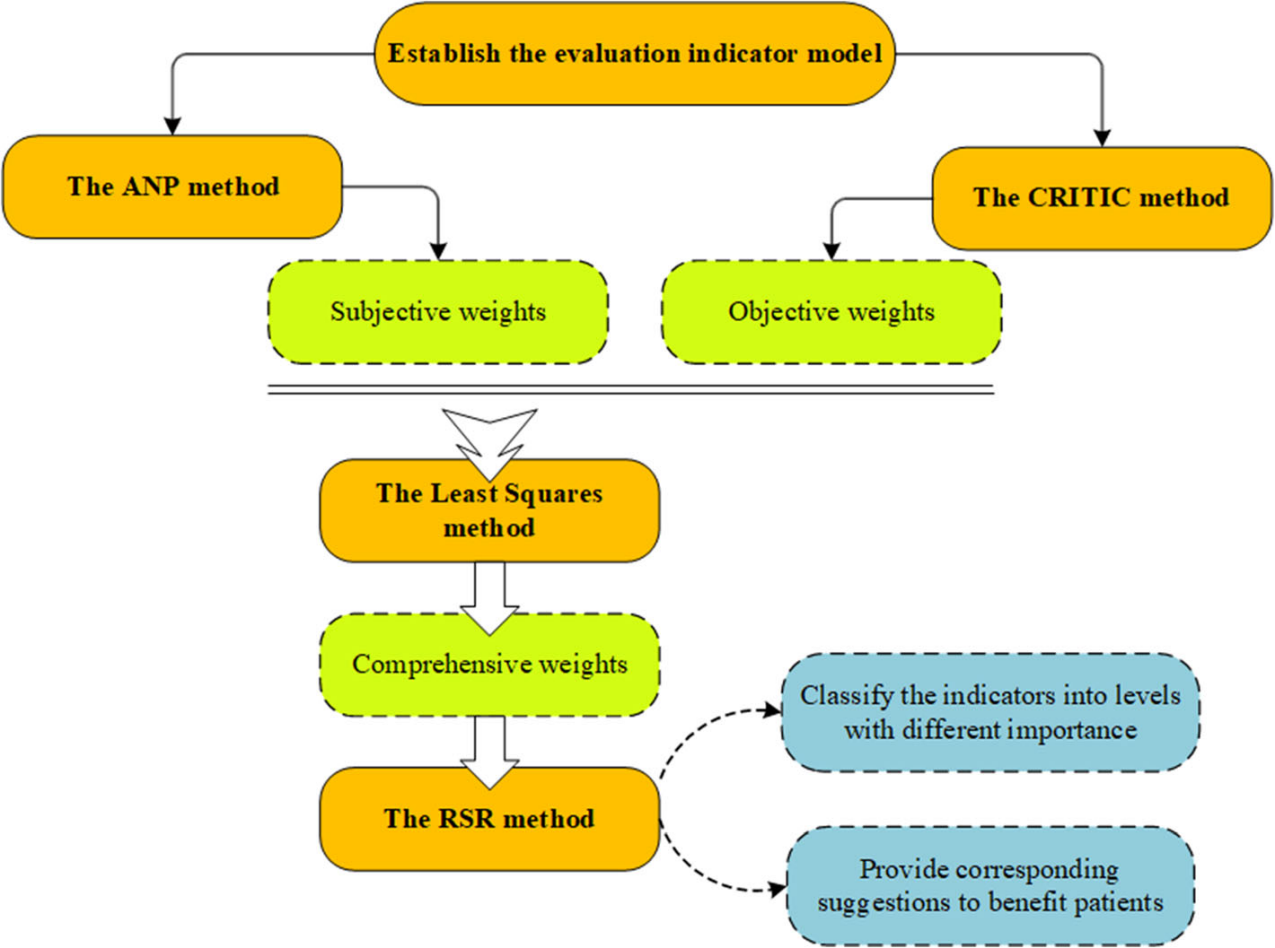
Methods framework. Methods framework: To figure out which social support indicators are more important for improving type 2 diabetes, we firstly established the evaluation indicator model in Section 2.1. Secondly, we used the ANP and CRITIC methods to determine their subjective and objective weights respectively. Thirdly, we achieved the fusion of subjective weight and objective weight by least square method, and obtained the comprehensive weights of indicators. Lastly, we used the RSR method to help health care providers understand the importance of different indicators more clearly by classifying them into different levels, and provide more effective social support

Figure 4 illustrated the framework of our study’s methods.

## Results

### Calculation of subjective weights

Firstly, we form a pairwise comparison matrix according to experts’ assessment of the importance of the three control criteria for social support. On this basis, we calculate the weighted values of each criterion to set and judge the CR of the matrix. Acceptable CR values must be less than 0.1. When CR ≥ 0.1, the judgment matrix should be properly corrected. The criterion comparison results are showed in Table 1.

**Table 1:** Criterion pairwise comparison matrix

Contribution	ES	IS	TS	Weights
ES	1	3	2	0.5396
IS	1/3	1	1/2	0.1634
TS	1/2	2	1	0.2970
CR = 0.0079				

It shows the criterion comparison results according to experts’ assessment of the importance of the three control criteria for social support

The weighting matrix of the three control criteria is as follows:

$$ {\omega}_1={\left(0.5396\kern0.5em 0.1634\kern0.5em 0.2970\right)}^{\mathrm{T}} $$

Then, we assume that there is no dependency or feedback relationship between the control criteria. Based on the scoring results of the panel of experts, we obtain the comparison matrix and weighting values for the sub-criteria, as shown in Additional file 5.

Next, we consider the relationship between the three control criteria and generate a dependency matrix with three factor sets as the criteria (for details, see Additional file 6). As a result, we get the following matrix:

$$ {\omega}_2=\left(\begin{array}{cccc}& ES& IS& TS\\ {} ES& 0& 0.25& 0.25\\ {} IS& 0.5& 0& 0.75\\ {} TS& 0.5& 0.75& 0\end{array}\right) $$

The weight of the interdependent control criteria (*ω*_3_) is calculated by multiplying the dependency matrix (*ω*_2_) and the weight matrix of the control criteria (*ω*_1_).

$$ {\omega}_3={\omega}_2\ast {\omega}_1={\left(0.1151\kern0.5em 0.4926\kern0.5em 0.3924\right)}^T $$

Moreover, we can use the assumptions shown in Table 2 to further obtain the weighted values of the sub-criteria.

**Table 2:** Sub-criterion pairwise comparison matrix

Criterion	Weights	Sub-criterion	Weights	Weights under the Overall Goal
ES	0.1151	E1	0.4667	0.0537
		E2	0.2979	0.0343
		E3	0.0849	0.0098
		E4	0.1504	0.0173
IS	0.4926	I1	0.1220	0.0601
		I2	0.5584	0.2750
		I3	0.3196	0.1574
TS	0.3924	T1	0.4554	0.1787
		T2	0.1409	0.0553
		T3	0.1409	0.0553
		T4	0.2628	0.1031

The weighted values of the sub-criteria were calculated by multiplying the dependency matrix and the weight matrix of the control criteria

The weight vector is

$$ {\omega}_{\mathrm{p}}={\left(0.0537\kern0.5em 0.0343\kern0.5em 0.0098\kern0.5em \begin{array}{cccc}0.0173& 0.0601& 0.2750& \begin{array}{cccc}0.1574& 0.1787& 0.0553& \begin{array}{cc}0.0553& 0.1031\end{array}\end{array}\end{array}\right)}^T $$

The dependency and feedback relationships provide a correlation between the sub-criteria and the two pairs of comparison matrices (for details, see Additional file 7). The resulting relative importance weights are used to generate the unweighted super-matrix (for details, see Additional file 8).

From the perspective of the expert opinions, the subjective weights of the 11 sub-criteria we finally obtained are:

$$ {\omega}_y={\omega}_u\ast {\omega}_p={\left(\begin{array}{cccc}\begin{array}{cccc}\begin{array}{cc}0.0615& 0.0587\end{array}& 0.0050& 0.0089& 0.0520\end{array}& 0.3409& 0.2052& 0.0955\end{array}\kern0.5em 0.0318\kern0.5em 0.0346\kern0.5em 0.1058\right)}^T $$

### Calculation of objective weights

The CRITIC data were obtained from a cohort of 2969 patients with type 2 diabetes mellitus. Based on the personal information recorded in medical records, we made a comprehensive analysis and found that: (1) the proportion of males is slightly higher than that of females, which accords with the gender distribution in China; (2) the age is mainly over 45 years old; (3) the proportion of family history of diabetes is more than one third. This information is basically consistent with the characteristics of people with type 2 diabetes. Then we began to use CRITIC method to calculate the objective weight of indicators. The relevant calculation formulas were shown in Additional file 9.

First, we used 2969 valid data sources collected through the questionnaire as the raw experimental data. These raw data are then processed according to Eq. (7) to obtain a normalized matrix *S*.

Second, the standard deviation *σ*_*i*_ of each row vector *S*_*i*_ is calculated to obtain the standard deviation vector *σ*.

$$ \sigma ={\left(\begin{array}{cccc}\begin{array}{cccc}\begin{array}{cc}0.3093& 0.3162\end{array}& 0.2624& 0.2674& 0.2992\end{array}& 0.3302& 0.3037& 0.3004\end{array}\kern0.5em 0.2788\kern0.5em 0.2766\kern0.5em 0.2839\right)}^T $$ Third, we calculate the linear correlation coefficient *r*_*ij*_ between the index *i* and the index *j* using MATLAB and consequently obtain the correlation matrix *R*.

Fourth, we calculate the amount of information contained in index *i* and expressed as *c*_*i*_ according to Eq. (8).

The result of the amount of information is

$$ C={\left(\begin{array}{cccc}\begin{array}{cccc}\begin{array}{cc}2.78& 3.14\end{array}& 2.12& 2.20& 2.79\end{array}& 3.74& 2.75& 3.10\end{array}\kern0.5em 3.06\kern0.5em 3.14\kern0.5em 2.30\right)}^T $$

Fifth, we normalize the vector *C* using Eq. (9).

Based on the valid data sources on social support collected from patients, the objective weights of the 11 sub-criteria obtained are:

$$ {\omega}_c={\left(\begin{array}{cccc}\begin{array}{cccc}\begin{array}{cc}0.0894& 0.1009\end{array}& 0.0681& 0.0708& 0.0898\end{array}& 0.1201& 0.0884& 0.0996\end{array}\kern0.5em 0.0983\kern0.5em 0.1009\kern0.5em 0.0739\right)}^T $$

### Calculation of comprehensive evaluation

As described in Sections 3.1 and 3.2, we can calculate the subjective weight vector *U* = (*u*_1_, *u*_2_, …, *u*_*m*_)^*T*^ and the objective weight vector *V* = (*v*_1_, *v*_2_, …, *v*_*m*_)^*T*^, separately. In this section, our main purpose is to integrate the above two weight vectors into a comprehensive weight vector *W* = (*w*_1_, *w*_2_, …, *w*_*m*_)^*T*^ using the least squares method, which considers both subjective and objective results. This is the standard optimization method for the approximate solution of the system, including the data set, where there is more data than the unknown. In the overall solution, the sum of the squares of the errors produced in the results of each individual data is minimized, which is the “least square” [39]. Therefore, the goal of the combined weighting method is to use the least squares equation to minimize the deviation between *U* and *V*.Based on the aforementioned analysis and computational process shown in Additional file 10, we can finally obtain the comprehensive weight values and shown in descending (Table 3).

**Table 3:** Comprehensive criterion pairwise comparison matrix

Sub-Criterion	ANP Weights	CRITIC Weights	Comprehensive Weights
I2	0.3409	0.1201	0.2305
I3	0.2052	0.0884	0.1468
T1	0.0955	0.0996	0.0975
T4	0.1058	0.0739	0.0899
E2	0.0587	0.1009	0.0798
E1	0.0615	0.0894	0.0755
I1	0.0520	0.0898	0.0709
T3	0.0346	0.1009	0.0677
T2	0.0318	0.0983	0.0651
E4	0.0089	0.0708	0.0398
E3	0.0050	0.0681	0.0366

It presents the subjective, objective and comprehensive weights of each criterion. And the indicator sets are then arranged in order of comprehensive weight values from largest to smallest

**Fig. 5:**
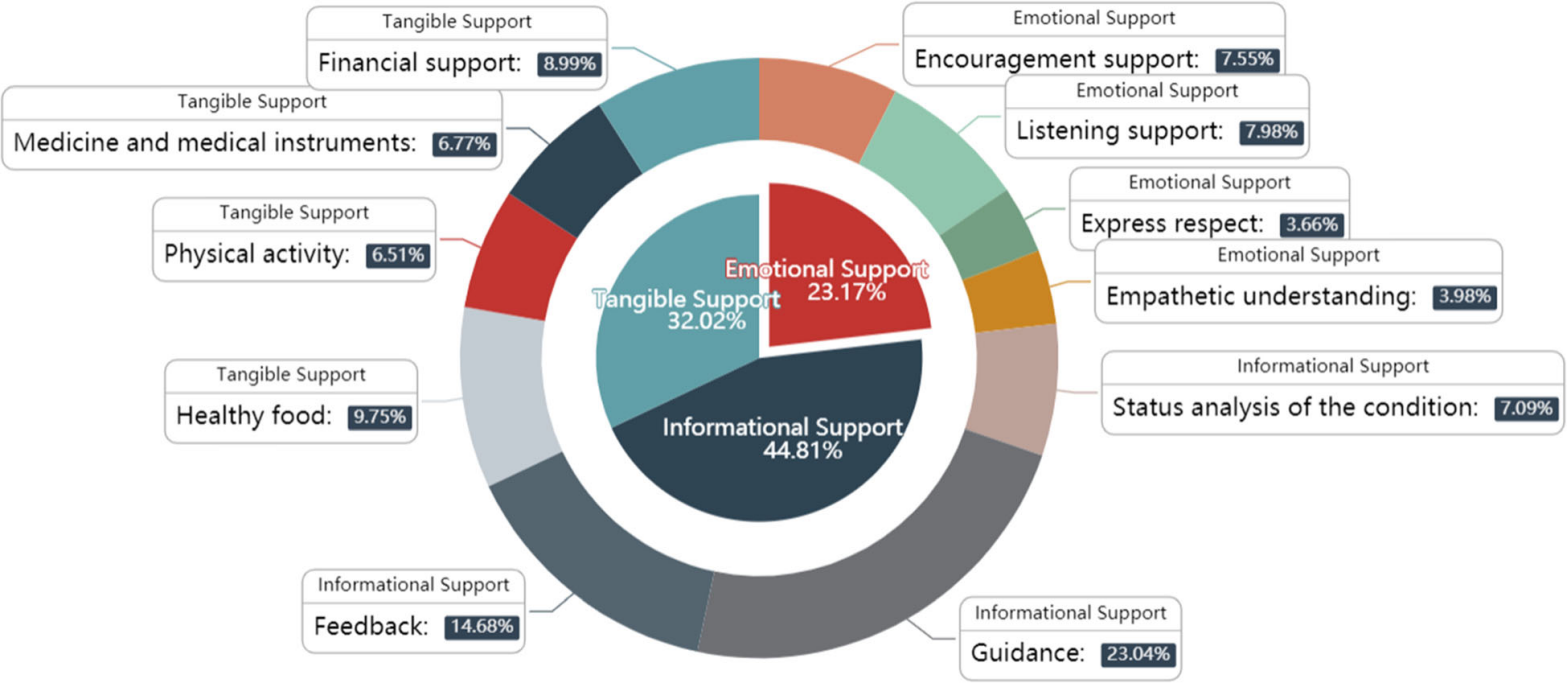
The weighs of indicators related to T2DM. The weighs of indicators related to T2DM: It visually demonstrates the impact of different social support indicators on self-management of type 2 diabetes. Informational support is the most important control criteria, followed by tangible support and emotional support. Moreover, guidance and feedback are two sub-criteria most relevant with T2DM self-management

In order to make the results clearer, we developed Fig. 5, in which the data was the percentage form of the comprehensive weights of these indicators. The larger the percentage value of the indicator, the more important it might be to improve self-management of type 2 diabetes. From the inner circle, we can see that the largest proportion of weights is informational support, occupying nearly half, whereas the smallest is emotional support at just 23.17%. Another sector, tangible support, accounts for 32.02%.

Overall, informational support dominates the given criteria indicators, which means that it plays the most important role in affecting T2DM. This may be related to the fact that diabetes is a chronic disease with a high demand for information, even greater than that of cancer and gastrointestinal or respiratory diseases [40]. However, there was some evidence that the information about diabetes management that patients receive is far less than what they need [40]. Therefore, it might be an effective way for health care providers to place informational support in a strategic position.

### Indicators’ classification based on the rank-sum ratio method

After calculating by the formulas in Additional file 11, we obtained the distribution of the RSR values of the indicators shown in Table 4.

**Table 4:** The distribution of the RSR values of the indicators

Indicator	RSR	f	f↓	R	$\overline{\boldsymbol{R}}$	***P***_***i***_	Y (Probit)
E3	0.0366	1	1	1	1	9.09%	3.6592
E4	0.0398	1	2	2	2	18.18%	4.0846
T2	0.0651	1	3	3	3	27.27%	4.3872
T3	0.0677	1	4	4	4	36.36%	4.6415
I1	0.0709	1	5	5	5	45.45%	4.8743
E1	0.0755	1	6	6	6	54.55%	5.1004
E2	0.0798	1	7	7	7	63.64%	5.3319
T4	0.0899	1	8	8	8	72.73%	5.5828
T1	0.0975	1	9	9	9	81.82%	5.8779
I3	0.1468	1	10	10	10	90.91%	6.2816
I2	0.2305	1	m = 11	11	11	97.73%^a^	6.8808

It shows the distribution of the RSR values of the indicators. It is worth noting that *P*_*m*_ is calculated by the equation of ${P}_m=\left(1-\frac{1}{4m}\right)\ast 100\%$

Ultimately, we classified the 11 indicators into three grades as shown in Table 5 according to the regulations on the critical probit values for different commonly used grade numbers (for details, see Additional file 12).

**Table 5:** The evaluation results based on the RSR method

Grade	Probit	Results
1	< 4.00	E3
2	[4.00,6.00)	E4, T2, T3, I1, E1, E2, T4, T1
3	≥6.00	I3, I2

It shows the evaluation results based on the RSR method and 11 sub-indicators are finally divided into three grades. The higher the grade of the indicators, the more important they are

From the analysis in Section 3.3, we can see that the 11 social support indicators have different degrees of impact on the self-management of type 2 diabetes, but it is unwise to pay the same attention to these indicators. On the one hand, it is hard to provide all-inclusive social support due to the limited time and energy of patients and health care providers. On the other hand, the impact of different indicators is different. The indicators that have less impact often have little effect on solving the problem. Focusing on the key indicators is guaranteed to solve the problem accurately and effectively.

Therefore, based on the RSR method, we divided the 11 social support indicators into three levels that have different influences on the self-management capability of type 2 diabetes and give patients and health care providers corresponding management strategies.

- High-sensitivity indicators (Grade 3): These include guidance (I2) and feedback (I3) with the highest weighs of 23.05 and 14.68%, respectively, which are the greatest influence on T2DM self-management. Patients and their health care providers should focus on these social support indicators, because any small improvements in these two indicators may yield substantial returns on self-management. Furthermore, health care providers must continue to monitor the patient’s condition over the long term and offer the targeted guidance and feedback the patient needs. This is because the course of T2DM is quite lengthy, the complications are complex, and in varying periods of the disease, the focus may differ, which means guidance and feedback needs will change when complications develop.
- Moderate-sensitivity indicators (Grade 2): This grade includes eight indicators: E4, T2, T3, I1, E1, E2, T4, and T1. All the tangible support indicators and most of the emotional support indicators are in this grade. They are less important than the Grade 3 indicators but still have a certain impact on T2DM self-management. If health care providers make good use of these moderate-sensitivity indicators as a supplement to the high-sensitivity indicators, it may be possible to further improve the self-management of type 2 diabetes. For example, arrange balanced diets and appropriate exercise for the patient and ensure their necessary medical and economic conditions. Attention should also be paid to changes in the patient’s mood, listening, encouraging, and expressing understanding to them.
- Low-sensitivity indicators (Grade 1): This grade only contains express respect (E3) with the lowest weight of 3.66%, suggesting that this indicator is not as important as those in Grades 2 and 3. We recommend that health care providers reduce their focus on it and place other indicators in a more prominent position.

All in all, we hope to help health care providers understand the importance of different indicators more clearly by stratifying them, and provide more effective social support.

## Discussion

In this paper, we proposed a weighting method that integrated subjective (ANP) and objective (CRITIC) evaluations to assess the effect of social support on improving self-management of type 2 diabetes.

Our main contributions are twofold:

- From the perspective of medical practice, type 2 diabetes is a painful long-term chronic disease, and its control depends on the patient’s own living habits and mental status to some extent. Social support plays an important role in this process, which means that health care providers need to understand how to maximize the full effectiveness of social support. Our research identified which social support indicators perform better in improving the self-management capacity of patients with type 2 diabetes. This clue is important for health care providers. By mastering this information, they will take note of the high-rewarding social support indicators and decide how to better provide social support. This will be a good theme for exploring personalized medicine. Furthermore, this means of social support analysis can also be applied to other diseases like type 2 diabetes, which has a long asymptomatic period, is easily affected by environmental factors, and requires daily care.
- From the perspective of methodology: (1) Establishing evaluation index system is seldom used in medical research. Based on full consideration of the social support multi-dimensionality, this study constructed the social support evaluation indicator system for type 2 diabetes for the first time, which filled the gap for medical research. (2) To our knowledge, this is the first study in which MCDM tools, specifically ANP and CRITIC, are used to evaluate the impact of social support on improving self-management of type 2 diabetes. They have more advantages than AHP and entropy weight method, which are commonly used in the MCDM field. For example, the ANP method has resolved the limitations of the AHP methodology by allowing feedback between the elements of the decision problem. Complex networks and interrelationships can be implemented using ANP [25]. In addition, the CRITIC method comprehensively determines the objective weight of the indicator by the size of the variation within the indicator and the conflict between the indicators, while the entropy weight method only considers the degree of variation of the indicator value. Through the integration of ANP and CRITIC, our study considered both the opinions of 15 authoritative experts and 2969 valid data sources on social support collected from KDM, which makes the results more reasonable and credible.

This study also has several limitations. The mechanism of social support effecting self-management is complex, and the selection of social support indicators that affect T2DM self-management may not be adequately and comprehensively considered. Because this study is the first to establish social support evaluation indicator system for T2DM self-management, it is difficult to find fully relevant literature and similar research methods in medical practice. However, this study provides a good basis for building a more scientific, accurate, and comprehensive evaluation indicator system in the future, and we will improve this point in our future research.

## Conclusion

This study aims to developing an integrated model to analyze which social support has more important impact on T2DM self-management. Based on literature review and inputs from experts, we firstly established an evaluation indicator system of social support consisting of three control criteria and eleven sub-criteria. Then we proposed the integrated model that combines ANP and CRITIC method to calculate the comprehensive weights of indicators to reflect the importance of them. Results show that the impact of different social support dimensions on the T2DM self-management varies widely. Information support, especially guidance and feedback, plays a relatively important role. Incorporating them into diabetes care plans has a great potential for improving behavioral and psychological health outcomes among people with T2DM. We hope that this research will help health care providers to figure out which social support is highly rewarding, so that they can fully play the positive role of social support when assisting patients and improve the self-management ability of T2DM patients.

## Supplementary information

**Additional file 1:** **Table S1.** Sub-criterion descriptions and references. It describes the meaning of 11 sub- criterion and the references.

**Additional file 2:** **Table S2.** Saaty’s 1–9 scale used in ANP. The scale is used to rate the importance between two criteria in the ANP method.

**Additional file 3:** Description: Experimental Informed Consent.

**Additional file 4:** **Table S4.1.** Social Support Measurement Questionnaire. The Social Support Measurement Questionnaire’s design considers the needs of our research itself and three widely used social support questionnaires: (1) The Inventory of Socially Supportive Behaviors (ISSB), (2) The Social Provisions Scale (SPS), and (3) The Medical Outcomes Study (MOS) Social Support Survey.

**Additional file 5:** **Table S5.1.** Pairwise comparison matrix on emotional support influence. According to the expert’s rating, we formed the pairwise comparison matrix on emotional support influence. And the weights of the four sub-criteria of emotional support are calculated. **Table S5.2.** Pairwise comparison matrix on informational support influence. According to the expert’s rating, we formed the pairwise comparison matrix on informational support influence. And the weights of the three sub-criteria of informational support are calculated. **Table S5.3.** Pairwise comparison matrix on tangible support influence. According to the expert’s rating, we formed the pairwise comparison matrix on tangible support influence. And the weights of the four sub-criteria of tangible support are calculated.

**Additional file 6:** **Table S6.1.** Under the overall objective criterion pairwise comparison matrix. It shows the three control criteria pairwise comparison results under the overall objective. **Table 6.2.** The inner dependency matrix of the factors with respect to other factors. Considering the interaction between the three control criteria, we form an inner dependency matrix of the factors with respect to other factors.

**Additional file 7:** **Table S7.1.** Pairwise comparison matrix on E1. According to the expert’s rating, we formed the pairwise comparison matrix on E1 (Encouragement support). **Table S7.2.** Pairwise comparison matrix on I2. According to the expert’s rating, we formed the pairwise comparison matrix on I2 (Guidance). **Table S7.3.** Pairwise comparison matrix on I3. According to the expert’s rating, we formed the pairwise comparison matrix on I3 (Feedback). **Table S7.4.** Pairwise comparison matrix on T1. According to the expert’s rating, we formed the pairwise comparison matrix on T1 (Healthy food). **Table S7.5.** Pairwise comparison matrix on T2. According to the expert’s rating, we formed the pairwise comparison matrix on T2 (Physical activity). **Table S7.6.** Pairwise comparison matrix on T3. According to the expert’s rating, we formed the pairwise comparison matrix on T3 (Medicine and medical instruments).

**Additional file 8:** **Table S8.** Unweighted super-matrix. The unweighted super-matrix is derived from the relative importance weights of each two sub-criteria.

**Additional file 9:** It describes the specific calculation steps of the CRITIC method.

**Additional file 10:** It describes the specific calculation steps of the least squares method.

**Additional file 11:** It describes the specific calculation steps of the RSR method.

**Additional file 12:** **Table S12.1.** Critical probit values for different commonly used grade numbers. It describes the regulations on the critical probit values for different commonly used grade numbers.

## Data Availability

The datasets used and/or analyzed during the current study are available from the corresponding author on reasonable request.

## Abbreviations

AHP: Analytic Hierarchy Process
ANP: Analytical Network Process
CI: Consistency Index
CR: Consistency Ratio
CRITIC: CRiteria Importance through Intercriteria Correlation
E1: Encouragement support
E2: Listening support
E3: Express respect
E4: Empathetic understanding
ES: Emotional Support
HbA1c: Glycosylated Hemoglobin
I1: Status analysis of the condition
I2: Guidance
I3: Feedback
IDF: International Diabetes Federation
IS: Informational Support
ISSB: Inventory of Socially Supportive Behaviors
KDM: Key Disease of Diabetes Mellitus Study
MCDM: Multi-Criteria Decision-Making
MOS: Medical Outcomes Study
RSR: Rank-Sum Ratio
SPS: Social Provisions Scale
T1: Healthy food
T2: Physical activity
T2DM: Type 2 Diabetes Mellitus
T3: Medicine and medical instruments
T4: Financial support
TS: Tangible Support
WHO: World Health Organization
