# Supplementary material for: An integrated model to evaluate the impact of social support on improving self-management of type 2 diabetes mellitus
Source: BMC Med Inform Decis Mak. 2019 Oct 22;19:197. doi: 10.1186/s12911-019-0914-9 (PMC6805520; doi:10.1186/s12911-019-0914-9)
Supplement: Supplementary file 1 — Additional file 1: Table S1. Sub-criterion descriptions and references. It describes the meaning of 11 sub- criterion and the references. [file 12911_2019_914_MOESM1_ESM.docx]

**Additional file 1 TableS1** Sub-criterion descriptions and references

| Control Criterion | Sub-Criterion | Sub-Criterion Descriptions | References |
| --- | --- | --- | --- |
| Emotional support | Encouragement support | It refers to whether someone gives you positive influence when you fail or want to give up and expresses his/her appreciation to you. | Aartsen (2015)  McPherson (2001)  Dam (2004) |
|  | Listening support | It refers to whether there is anyone you can rely on when you want to talk. | Aartsen (2015)  McPherson (2001) |
|  | Express respect | It means expressing via your behavior commitment and reassuring the person that he or she is a valuable individual who is cared for. | Aartsen (2015)  McPherson (2001) |
|  | Empathetic understanding | It refers to whether someone is understanding and cares about your pain or misfortune. | Dam (2004)  Taylor (1999)  Aartsen (2015) |
| Informational support | Status analysis of the condition | It refers to the interpretation and analysis of the patient's current condition. | Dam (2004)  Taylor (1999) |
|  | Guidance | It refers to helping a person understand better lifestyle adaptations and what resources and coping strategies may be mastered to deal with type 2 diabetes. This guidance can be spread in social relationships through language and action, often by making suggestions. | Siegel (1993)  Dam (2004)  Taylor (1999) |
|  | Feedback | It refers to the effectiveness of correct advice on the management of type 2 diabetes after you have completed the physical examination or self-description. | Dam (2004)  Taylor (1999)  Aartsen (2015) |
| Tangible support | Healthy food | It refers to the daily use of people to scientifically and rationally control blood sugar and reduce the impact of diabetes on the health of the various dietary activities. |  |
|  | Physical activity | It refers to the extent to which people in their social network were supportive of physical activity as well as whether there were individuals who participated in physical activity with them. | Krause (1999)  Steward (1999)  Aartsen (2015) |
|  | Medicine and medical instruments | It refers to whether you can obtain a health care product, medications, and instruments, and whether you can reach the medical and medical institutions, that is, the level of medical resources you can obtain. | Krause (1999)  Steward (1999)  Dam, et al. (2004) |
|  | Financial support | When you need financial resources to control or treat type 2 diabetes or even to prevent its occurrence, the financial amount you can obtain, and the time you need to obtain finance. The greater the amount or the shorter the time, the more support you receive. | Krause (1999)  Steward (1999) |
