# Supplementary material for: An integrated model to evaluate the impact of social support on improving self-management of type 2 diabetes mellitus
Source: BMC Med Inform Decis Mak. 2019 Oct 22;19:197. doi: 10.1186/s12911-019-0914-9 (PMC6805520; doi:10.1186/s12911-019-0914-9)
Supplement: Supplementary file 2 — Additional file 2: Table S2. Saaty’s 1–9 scale used in ANP. The scale is used to rate the importance between two criteria in the ANP method. [file 12911_2019_914_MOESM2_ESM.docx]

**Additional file 2 Table S2 Saaty's 1-9 scale used in ANP**

| Intensity of Importance | Definition |
| --- | --- |
| 1 | Equal importance |
| 3 | Moderate importance |
| 5 | Strong importance |
| 7 | Very strong importance |
| 9 | Absolute importance |
| 2,4,6,8 | Used to express intermediate values |
