# Supplementary material for: An integrated model to evaluate the impact of social support on improving self-management of type 2 diabetes mellitus
Source: BMC Med Inform Decis Mak. 2019 Oct 22;19:197. doi: 10.1186/s12911-019-0914-9 (PMC6805520; doi:10.1186/s12911-019-0914-9)
Supplement: Supplementary file 3 — Additional file 3: Description: Experimental Informed Consent. [file 12911_2019_914_MOESM3_ESM.docx]

**Additional file 3 Experimental Informed Consent**

**Research background introduction:**

You are about to participate in a study organized by Hefei University of Technology to prove which social support has greater impact on self-management of Type 2 Diabetes Mellitus (T2DM). It will take you about 15 minutes. As you meet the requirements of the subjects, you are invited to participate in this experiment.

This informed consent will provide some information that will help you decide whether to participate in the study or not. Your participation in this study is voluntary and has been examined by the Medical Ethics Committee of this research institution. If you agree to join the study, please read the following instructions in detail:

**1. Research purposes:**

The purpose of this study is to analyze which social support has more significant impact on T2DM self-management, and provide reasonable suggestions to health care providers on how to effectively play the role of social support.

**2. Research process:**

We need to know your basic information from your electronic medical record and invite you to fill in a questionnaire. Please answer according to your actual situation.

**3. Study risk and discomfort**

Participating in the experiment, you may have a slight dizziness, you will get some data related to you, or expose your sensitive information and emotions, of course, we will keep it strictly confidential.

**5.** **Privacy issues:**

If you decide to participate in this research, your personal data will be kept strictly confidential. We will not disclose it to third parties unless you allow it. In order to ensure the authenticity of the experiment, you must provide your authentic information. Your research materials are available only to researchers. If necessary, members of the government administration or the ethics review committee are required to have access to your personal data in the research institute.

For the confidential information of this experiment, you must also abide by the principle of confidentiality and not disclose important information to third parties.

If you have any questions related to this study, or have any discomfort or injury in the course of the study, or have questions about the rights and interests of the participants in this study, you can contact the head of the experiment xxx, telephone number: xxxxxxxxxxx.

**Informed consent signature:**

I have read this informed consent carefully, and the researcher has explained the purpose, process, risk and privacy issues of the experiment to me in detail, and answered the questions I asked. I have learned about the experiment, and I volunteered to participate in it.

**Signature:**

**Date:**
