# Supplementary material for: An integrated model to evaluate the impact of social support on improving self-management of type 2 diabetes mellitus
Source: BMC Med Inform Decis Mak. 2019 Oct 22;19:197. doi: 10.1186/s12911-019-0914-9 (PMC6805520; doi:10.1186/s12911-019-0914-9)
Supplement: Supplementary file 4 — Additional file 4: Table S4.1. Social Support Measurement Questionnaire. The Social Support Measurement Questionnaire’s design considers the needs of our research itself and three widely used social support questionnaires: (1) The Inventory of Socially Supportive Behaviors (ISSB), (2) The Social Provisions Scale (SPS), and (3) The Medical Outcomes Study (MOS) Social Support Survey. [file 12911_2019_914_MOESM4_ESM.docx]

**Additional file 4 Table S4** Social Support Measurement Questionnaire

| Indicator | Number | Items | Option | | | | |
| --- | --- | --- | --- | --- | --- | --- | --- |
|  |  |  | Strongly Agree | Agree | Not Sure | Disagree | Strongly Disagree |
| Encouragement support | 1 | Somebody who encourages me to maintain the diet recommended by my physician or nutritionist. |  |  |  |  |  |
|  | 2 | Somebody who joked and kidded to try to cheer you up. |  |  |  |  |  |
|  | 3 | Somebody who encourages me to perform self-monitoring of blood glucose independently. |  |  |  |  |  |
| Listening support | 4 | Listened to you talk about your private feelings. |  |  |  |  |  |
|  | 5 | I have friends/relatives who will definitely take time to listen if I need someone to talk to. |  |  |  |  |  |
|  | 6 | If I'm very depressed, I know who I can turn to. |  |  |  |  |  |
| Express respect | 7 | Express respect for something you did well. |  |  |  |  |  |
|  | 8 | No one discriminates against you because you are sick. |  |  |  |  |  |
|  | 9 | Express respect for the pain or anxiety you are complaining about. |  |  |  |  |  |
| Empathetic understanding | 10 | Told you how he/she felt in a situation that was similar to yours. |  |  |  |  |  |
|  | 11 | Someone expressed sympathy for your condition. |  |  |  |  |  |
|  | 12 | Someone understands your pain or anxiety. |  |  |  |  |  |
| Status analysis of the condition | 13 | Gave you some information to help you understand a situation that you were in. |  |  |  |  |  |
|  | 14 | Said things that made your situation clearer and easier to understand. |  |  |  |  |  |
|  | 15 | Told you what to expect in a situation that was about to happen. |  |  |  |  |  |
| Guidance | 16 | Suggested some action that you should take. |  |  |  |  |  |
|  | 17 | Assisted you in setting a goal for yourself. |  |  |  |  |  |
|  | 18 | Gave you some information on how to do something. |  |  |  |  |  |
| Feedback | 19 | Gave you feedback on how you were doing without saying it was good or bad. |  |  |  |  |  |
|  | 20 | Agreed that what you wanted to do was right. |  |  |  |  |  |
|  | 21 | Helped you understand why you did not do something well. |  |  |  |  |  |
| Healthy food | 22 | Somebody who buys me the necessary ingredients to cook appropriate foods for diabetics. |  |  |  |  |  |
|  | 23 | Somebody who cooks me appropriate foods for a diabetic patient. |  |  |  |  |  |
|  | 24 | Somebody who eats the foods that I can eat so that I do not have any temptation and can stay on my diet. |  |  |  |  |  |
| Physical activity | 25 | Someone arranges exercise programs for you. |  |  |  |  |  |
|  | 26 | Someone provides you with the fitness equipment you need. |  |  |  |  |  |
|  | 27 | Someone prepares a venue or environment for you to exercise. |  |  |  |  |  |
| Medicine and medical instruments | 28 | Somebody who checks all of the necessary equipment to perform self-monitoring of blood glucose. |  |  |  |  |  |
|  | 29 | Somebody who helps me monitor my blood glucose by glucometer when I am not strong enough. |  |  |  |  |  |
|  | 30 | Somebody who performs daily foot care for me when I am not strong enough. |  |  |  |  |  |
| Financial support | 31 | Somebody who pays the cost of joining a gym or buying exercise equipment. |  |  |  |  |  |
|  | 32 | Somebody who pays the cost of medicine. |  |  |  |  |  |
|  | 33 | If you need some extra help financially, you can count on someone to help you. |  |  |  |  |  |
