# Supplementary material for: An integrated model to evaluate the impact of social support on improving self-management of type 2 diabetes mellitus
Source: BMC Med Inform Decis Mak. 2019 Oct 22;19:197. doi: 10.1186/s12911-019-0914-9 (PMC6805520; doi:10.1186/s12911-019-0914-9)
Supplement: Supplementary file 5 — Additional file 5: Table S5.1. Pairwise comparison matrix on emotional support influence. According to the expert’s rating, we formed the pairwise comparison matrix on emotional support influence. And the weights of the four sub-criteria of emotional support are calculated. Table S5.2. Pairwise comparison matrix on informational support influence. According to the expert’s rating, we formed the pairwise comparison matrix on informational support influence. And the weights of the three sub-criteria of informational support are calculated. Table S5.3. Pairwise comparison matrix on tangible support influence. According to the expert’s rating, we formed the pairwise comparison matrix on tangible support influence. And the weights of the four sub-criteria of tangible support are calculated. [file 12911_2019_914_MOESM5_ESM.docx]

**Additional file 5: Table S5.** Pairwise comparison matrix on emotional support influence.

| ES | E1 | E2 | E3 | E4 | Weights |
| --- | --- | --- | --- | --- | --- |
| E1 | 1 | 2 | 5 | 3 | 0.4667 |
| E2 | 1/2 | 1 | 3 | 2 | 0.2979 |
| E3 | 1/5 | 1/3 | 1 | 1/2 | 0.0849 |
| E4 | 1/3 | 1/2 | 2 | 1 | 0.1504 |
| CR=0.0569 | | | | | |

**Table 5.2** Pairwise comparison matrix on informational support influence.

| IS | I1 | I2 | I3 | Weights |
| --- | --- | --- | --- | --- |
| I1 | 1 | 1/4 | 1/3 | 0.1220 |
| I2 | 4 | 1 | 2 | 0.5584 |
| I3 | 3 | 1/2 | 1 | 0.3196 |
| CR=0.0158 | | | | |

**Table 5.3** Pairwise comparison matrix on tangible support influence.

| TS | T1 | T2 | T3 | T4 | Weights |
| --- | --- | --- | --- | --- | --- |
| T1 | 1 | 3 | 3 | 2 | 0.4554 |
| T2 | 1/3 | 1 | 1 | 1/2 | 0.1409 |
| T3 | 1/3 | 1 | 1 | 1/2 | 0.1409 |
| T4 | 1/2 | 2 | 2 | 1 | 0.2628 |
| CR=0.0038 | | | | | |
