# Supplementary material for: An integrated model to evaluate the impact of social support on improving self-management of type 2 diabetes mellitus
Source: BMC Med Inform Decis Mak. 2019 Oct 22;19:197. doi: 10.1186/s12911-019-0914-9 (PMC6805520; doi:10.1186/s12911-019-0914-9)
Supplement: Supplementary file 6 — Additional file 6: Table S6.1. Under the overall objective criterion pairwise comparison matrix. It shows the three control criteria pairwise comparison results under the overall objective. Table 6.2. The inner dependency matrix of the factors with respect to other factors. Considering the interaction between the three control criteria, we form an inner dependency matrix of the factors with respect to other factors. [file 12911_2019_914_MOESM6_ESM.docx]

**Additional file 6.**

**Table 6.1** Under the overall objective criterion pairwise comparison matrix.

| Contribution | ES | IS | TS | Weights |
| --- | --- | --- | --- | --- |
| ES | 1 | 3 | 2 | 0.5396 |
| IS | 1/3 | 1 | 1/2 | 0.1634 |
| TS | 1/2 | 2 | 1 | 0.2970 |
| CR=0.0079 | | | | |

**Table 6.2** The inner dependency matrix of the factors with respect to other factors.

| ES | IS | TS | Weights |
| --- | --- | --- | --- |
| IS | 1 | 1 | 0.50 |
| TS | 1 | 1 | 0.50 |
| CR=0.00 | | | |
| IS | ES | TS | Weights |
| ES | 1 | 1/3 | 0.2500 |
| TS | 3 | 1 | 0.7500 |
| CR=0.00 | | | |
| TS | ES | IS | Weights |
| ES | 1 | 1/3 | 0.2500 |
| IS | 3 | 1 | 0.7500 |
| CR=0.00 | | | |
