# Supplementary material for: An integrated model to evaluate the impact of social support on improving self-management of type 2 diabetes mellitus
Source: BMC Med Inform Decis Mak. 2019 Oct 22;19:197. doi: 10.1186/s12911-019-0914-9 (PMC6805520; doi:10.1186/s12911-019-0914-9)
Supplement: Supplementary file 7 — Additional file 7: Table S7.1. Pairwise comparison matrix on E1. According to the expert’s rating, we formed the pairwise comparison matrix on E1 (Encouragement support). Table S7.2. Pairwise comparison matrix on I2. According to the expert’s rating, we formed the pairwise comparison matrix on I2 (Guidance). Table S7.3. Pairwise comparison matrix on I3. According to the expert’s rating, we formed the pairwise comparison matrix on I3 (Feedback). Table S7.4. Pairwise comparison matrix on T1. According to the expert’s rating, we formed the pairwise comparison matrix on T1 (Healthy food). Table S7.5. Pairwise comparison matrix on T2. According to the expert’s rating, we formed the pairwise comparison matrix on T2 (Physical activity). Table S7.6. Pairwise comparison matrix on T3. According to the expert’s rating, we formed the pairwise comparison matrix on T3 (Medicine and medical instruments). [file 12911_2019_914_MOESM7_ESM.docx]

**Additional file 7.**

**Table 7.1** Pairwise comparison matrix on E1.

| E1 | I2 | T1 | T2 | T3 | Weights |
| --- | --- | --- | --- | --- | --- |
| I2 | 1 | 4 | 4 | 3 | 0.5385 |
| T1 | 1/4 | 1 | 1 | 1/2 | 0.1210 |
| T2 | 1/4 | 1 | 1 | 1/2 | 0.1210 |
| T3 | 1/3 | 2 | 2 | 1 | 0.2196 |
| CR=0.0076 | | | | | |

**Table 7.2** Pairwise comparison matrix on I2.

| I2 | I3 | T4 | Weights |
| --- | --- | --- | --- |
| I3 | 1 | 3 | 0.7500 |
| T4 | 1/3 | 1 | 0.2500 |
| CR=0.00 | | | |

**Table 7.3** Pairwise comparison matrix on I3

| I3 | E2 | I2 | Weights |
| --- | --- | --- | --- |
| E2 | 1 | 1/2 | 0.3333 |
| I2 | 2 | 1 | 0.6667 |
| CR=0.00 | | | |

**Table 7.4** Pairwise comparison matrix on T1.

| T1 | E1 | I1 | I2 | T4 | Weights |
| --- | --- | --- | --- | --- | --- |
| E1 | 1 | 2 | 1/2 | 3 | 0.2720 |
| I1 | 1/2 | 1 | 1/3 | 2 | 0.1570 |
| I2 | 2 | 3 | 1 | 5 | 0.4829 |
| T4 | 1/3 | 1/2 | 1/5 | 1 | 0.0882 |
| CR=0.0054 | | | | | |

**Table 7.5** Pairwise comparison matrix on T2.

| T2 | E1 | I1 | I2 | Weights |
| --- | --- | --- | --- | --- |
| E1 | 1 | 3 | 1/2 | 0.3090 |
| I1 | 1/3 | 1 | 1/5 | 0.1095 |
| I2 | 2 | 5 | 1 | 0.5816 |
| CR=0.0032 | | | | |

**Table 7.6** Pairwise comparison matrix on T3.

| T3 | I1 | I2 | T4 | Weights |
| --- | --- | --- | --- | --- |
| I1 | 1 | 1/4 | 1/3 | 0.1220 |
| I2 | 4 | 1 | 2 | 0.5584 |
| T4 | 1/3 | 1/2 | 1 | 0.3196 |
| CR=0.0158 | | | | |
