# Supplementary material for: An integrated model to evaluate the impact of social support on improving self-management of type 2 diabetes mellitus
Source: BMC Med Inform Decis Mak. 2019 Oct 22;19:197. doi: 10.1186/s12911-019-0914-9 (PMC6805520; doi:10.1186/s12911-019-0914-9)
Supplement: Supplementary file 8 — Additional file 8: Table S8. Unweighted super-matrix. The unweighted super-matrix is derived from the relative importance weights of each two sub-criteria. [file 12911_2019_914_MOESM8_ESM.docx]

**Additional file 8.**

**Table 8.1** Unweighted super-matrixes.

| $\boldsymbol{\omega}_{\boldsymbol{u}}$ | E1 | E2 | E3 | E4 | I1 | I2 | I3 | T1 | T2 | T3 | T4 |
| --- | --- | --- | --- | --- | --- | --- | --- | --- | --- | --- | --- |
| E1 | 1 | 0 | 0 | 0 | 0 | 0 | 0 | 0.272 | 0.3090 | 0 | 0 |
| E2 | 0 | 1 | 1 | 1 | 0 | 0 | 0.3333 | 0 | 0 | 0 | 0 |
| E3 | 0 | 0 | 1 | 0 | 0 | 0 | 0 | 0 | 0 | 0 | 0 |
| E4 | 0 | 0 | 0 | 1 | 0 | 0 | 0 | 0 | 0 | 0 | 0 |
| I1 | 0 | 0 | 0 | 0 | 1 | 0 | 0 | 0.1570 | 0.1095 | 0.1220 | 0 |
| I2 | 0.5385 | 0 | 0 | 0 | 0 | 1 | 0.6667 | 0.4829 | 0.5816 | 0.5584 | 1 |
| I3 | 0 | 1 | 0 | 0 | 0 | 0.75 | 1 | 0 | 0 | 0 | 0 |
| T1 | 0.1210 | 0 | 0 | 0 | 0 | 0 | 0 | 1 | 0 | 0 | 0 |
| T2 | 0.1210 | 0 | 0 | 0 | 0 | 0 | 0 | 0 | 1 | 0 | 0 |
| T3 | 0.2196 | 0 | 0 | 0 | 0 | 0 | 0 | 0 | 0 | 1 | 0 |
| T4 | 0 | 0 | 0 | 0 | 0 | 0.25 | 0 | 0.0882 | 0 | 0.3196 | 1 |
