# Supplementary material for: An integrated model to evaluate the impact of social support on improving self-management of type 2 diabetes mellitus
Source: BMC Med Inform Decis Mak. 2019 Oct 22;19:197. doi: 10.1186/s12911-019-0914-9 (PMC6805520; doi:10.1186/s12911-019-0914-9)
Supplement: Supplementary file 9 — Additional file 9: It describes the specific calculation steps of the CRITIC method. [file 12911_2019_914_MOESM9_ESM.docx]

**Additional file 9.**

| $S_{ij}=\frac{x_{ij}-x_{i-\min}}{x_{i-\max}-x_{i-\min}}$ | (7) |
| --- | --- |

$S_{ij}$ is the normalized value and $x_{ij}$ is the original value of the variable$j$. $x_{i-\max}$ and $x_{i-\min}$represent the maximum and minimum values of the index $i$, respectively.

| $c_{i}=\sigma_{i}\sum_{j=1}^{n} (1-r_{ij})$ | (8) |
| --- | --- |

where $c_{i}$represents the amount of information contained in index $i$ and $\sigma_{i}$ is the standard deviation of index $i$. In addition, $r_{ij}$ is the correlation coefficient between index $i$ and index $j$ and $n$ is the index number.

| $\omega_{i}=c_{i}/\sum_{j=1}^{n} c_{j}$ | (9) |
| --- | --- |

where $\omega_{i}$ is the weight of index $i$, $c_{i}$ is the amount of information contained in index $i$, and $n$ represents the index number.
