# Supplementary material for: An integrated model to evaluate the impact of social support on improving self-management of type 2 diabetes mellitus
Source: BMC Med Inform Decis Mak. 2019 Oct 22;19:197. doi: 10.1186/s12911-019-0914-9 (PMC6805520; doi:10.1186/s12911-019-0914-9)
Supplement: Supplementary file 10 — Additional file 10: It describes the specific calculation steps of the least squares method. [file 12911_2019_914_MOESM10_ESM.docx]

**Additional file 10.**

the object function is constructed as follows:

$\min H(w)=\sum_{i=1}^{n} \sum_{j=1}^{m} \left\{\begin{matrix} \left[(u_{j}-w_{j})z_{ij}\right]^{2} & + & \left[(v_{j}-w_{j})z_{ij}\right]^{2} \end{matrix}\right\}$ (10)

subject to

$\sum_{j=1}^{m} w_{j}=1,\begin{matrix} \end{matrix}w_{j}\geq0\begin{matrix} \end{matrix}(j=1,2,\cdots,m)$ (11)

where $z_{ij}$denotes the normalized indicator value.

To solve the object function (10), the Lang range function is introduced and defined as follows:

$$L=\sum_{i=1}^{n} \sum_{j=1}^{m} \left\{\left[\left(u_{j}-w_{j}\right)z_{ij}\right]^{2}+\left[\left(v_{j}-w_{j}\right)z_{ij}\right]^{2}+4\lambda\left(\sum_{j=1}^{m} w_{j}-1\right)\right\}$$

Let

$$\begin{matrix} \frac{\partial L}{\partial w_{j}}=-\sum_{i=1}^{n} 2\left(u_{j}+v_{j}-2w_{j}\right)z_{ij}^{2}+4\lambda=0\left(j=1,2,...,m\right) \\ \frac{\partial L}{\partial\lambda}=4\left(\sum_{j=1}^{m} w_{j}-1\right)=0 \end{matrix}$$

Alternatively, it can be represented by the following matrix Eq. (12)

| $\left[\begin{matrix} A & e \\ e^{T} & 0 \end{matrix}\right]\left[\begin{matrix} W \\ \lambda\end{matrix}\right]=\left[\begin{matrix} B \\ 1 \end{matrix}\right]$ |
| --- |

Where,

|  |  | (12) |
| --- | --- | --- |

By solving Eq. (13), the combined weight vector $W$ is calculated as the following Eq. (13)

|  | $W=A^{-1}\left[B+\frac{1-e^{T}A^{-1}B}{e^{T}A^{-1}e}e\right]$ | (13) |  |
| --- | --- | --- | --- |
