# Supplementary material for: An integrated model to evaluate the impact of social support on improving self-management of type 2 diabetes mellitus
Source: BMC Med Inform Decis Mak. 2019 Oct 22;19:197. doi: 10.1186/s12911-019-0914-9 (PMC6805520; doi:10.1186/s12911-019-0914-9)
Supplement: Supplementary file 11 — Additional file 11: It describes the specific calculation steps of the RSR method. [file 12911_2019_914_MOESM11_ESM.docx]

**Additional file 11.**

First, we replace the $RSR_{i}$ value with the value of the comprehensive weights. The indicator sets are then arranged in order of $RSR_{i}$ values from smallest to largest. Finally, the frequencies $f_{i}$ and cumulative frequencies $f\downarrow_{i}$ are listed and the percentiles $P{}_{i}$ is calculated according to the formula (14).

| $f\downarrow_{i}=i$ | (14) |
| --- | --- |
| $P_{i}=\frac{f\downarrow}{m},i=1,2,...,m-1$ |  |

Then determine the corresponding probit $Y{}_{i}$
