# Supplementary material for: An integrated model to evaluate the impact of social support on improving self-management of type 2 diabetes mellitus
Source: BMC Med Inform Decis Mak. 2019 Oct 22;19:197. doi: 10.1186/s12911-019-0914-9 (PMC6805520; doi:10.1186/s12911-019-0914-9)
Supplement: Supplementary file 12 — Additional file 12: Table S12.1. Critical probit values for different commonly used grade numbers. It describes the regulations on the critical probit values for different commonly used grade numbers. [file 12911_2019_914_MOESM12_ESM.docx]

**Additional file 12.**

**Table 12.1** Critical probit values for different commonly used grade numbers

| Grade numbers | Probit |
| --- | --- |
| 3 | 4.00,6.00 |
| 4 | 3.50,5.00,6.50 |
| 5 | 3.20,4.40,5.60,6.80 |
| 6 | 3.00,4.00,5.00,6.00,7.00 |
| 7 | 2.86,3.72,4.57,5.44,6.28,7.14 |
